# Graph convolutional network for fMRI analysis based on connectivity neighborhood

**DOI:** 10.1162/netn_a_00171

**Published:** 2021-02-01

**Authors:** Lebo Wang, Kaiming Li, Xiaoping P. Hu

**Affiliations:** Department of Electrical and Computer Engineering, University of California, Riverside, Riverside, CA, USA; Department of Bioengineering, University of California, Riverside, Riverside, CA, USA; Department of Electrical and Computer Engineering, University of California, Riverside, Riverside, CA, USA; Department of Bioengineering, University of California, Riverside, Riverside, CA, USA

**Keywords:** Functional connectivity, Deep learning, Graph convolutional network, Connectivity-based neighborhood

## Abstract

There have been successful applications of deep learning to functional magnetic resonance imaging (fMRI), where fMRI data were mostly considered to be structured grids, and spatial features from Euclidean neighbors were usually extracted by the convolutional neural networks (CNNs) in the computer vision field. Recently, CNN has been extended to graph data and demonstrated superior performance. Here, we define graphs based on functional connectivity and present a connectivity-based graph convolutional network (cGCN) architecture for fMRI analysis. Such an approach allows us to extract spatial features from connectomic neighborhoods rather than from Euclidean ones, consistent with the functional organization of the brain. To evaluate the performance of cGCN, we applied it to two scenarios with resting-state fMRI data. One is individual identification of healthy participants and the other is classification of autistic patients from normal controls. Our results indicate that cGCN can effectively capture functional connectivity features in fMRI analysis for relevant applications.

## INTRODUCTION

In recent years, there has been increasing interest in studying the brain via resting-state functional magnetic resonance imaging (rs-fMRI). Without any task or external stimulus, rs-fMRI can capture the spontaneous brain activity that reflects the functional organization of the brain. Based on rs-fMRI data, functional connectivity (FC) has been calculated using correlations between spatially distant regions within/across functional networks (Rogers, Morgan, Newton, & Gore, 2007; van den Heuvel & Pol, 2010). FC is believed to reflect the functional organization of the brain and has been used as a fingerprint to identify individuals (Finn et al., 2015). Alterations in FC have been associated with psychiatric disorders (Bullmore & Sporns, 2009; Greicius, Supekar, Menon, & Dougherty, 2009; van den Heuvel & Pol, 2010), demonstrating FC’s potential as biomarkers in clinical neuroscience. Various methods have been introduced to analyze the spatial FC pattern with fMRI data, including correlation-based approaches (Rogers et al., 2007; van den Heuvel & Pol, 2010), graph-based methods (Keown et al., 2017; Lee, Smyser, & Shimony, 2013; Wang, Zuo, & He, 2010), and matrix factorization techniques (Andersen, Gash, & Avison, 1999; van de Ven, Formisano, Prvulovic, Roeder, & Linden, 2004). The temporal pattern of FC has also been analyzed with several methods, including sliding-window analysis (Sakoğlu et al., 2010), time-frequency analysis (Chang & Glover, 2010), and the Gaussian hidden Markov model (Chen, Langley, Chen, & Hu, 2016). With the increasing availability of large fMRI datasets (Craddock et al., 2013; Miller et al., 2016; Van Essen et al., 2013), deep learning–based methods are becoming more widely used in fMRI analysis.

### Deep Learning on fMRI Data

Deep learning architectures, especially convolutional neural networks (CNN), have achieved remarkable performance in many applications, including image classification (He, Zhang, Ren, & Sun, 2016), image segmentation (Ronneberger, Fischer, & Brox, 2015), and machine translation (Vaswani et al., 2017). These applications mostly utilized automatic feature extraction via an end-to-end training paradigm on structured data (e.g., images and text). Similar strategies were extended to neuroimaging data, and promising results were obtained in several applications, including Alzheimer patient classification (Sarraf & Tofighi, 2016), subcortical brain structure segmentation (Dolz, Desrosiers, & Ayed, 2018), and segmentation of longitudinal structural MRI (Gao et al., 2018). However, there are several limitations to model fMRI data on the perspective of image grids: (a) It is computationally intensive to deal with voxelwise fMRI data via CNN models; (b) Brain activity occurs mostly in cortical and subcortical structures, making convolutions on white matter unnecessary; (c) The fMRI time course from a single voxel is usually very noisy; and (d) spatial features may be confined to a small neighborhood in the Euclidean space, especially in shallow CNN models. Therefore, it is of great necessity to build deep learning models that are more appropriate for the organization of the brain and that can efficiently extract connectomic features beyond a single voxel and its Euclidean neighborhood.

### Deep Learning on FC Matrix

To reduce the spatial complexity and noise in individual voxel data, many studies have turned to approaches based on regions of interest (ROIs). By calculating the correlation coefficients between pairs of ROIs based on the whole scan, the ROI-derived FC matrix reveals the temporal correlation pattern of ROIs. Because of the grid structure of the 2D matrix, the FC matrix shows great compatibility with traditional deep learning models. Therefore, the FC matrix has been directly adopted as inputs of deep learning approaches in several studies. For instance, Suk, Wee, Lee, and Shen (2016) introduced a deep learning architecture to investigate functional dynamics for mild cognitive impairment. Heinsfeld, Franco, Craddock, Buchweitz, and Meneguzzi (2018) identified autism spectrum disorder (ASD) based on autoencoders with the FC matrix as input data. However, the FC matrix describes the linear temporal relationship between ROIs but does not account for the rich temporal dynamic information in the time courses. Our prior work with recurrent neural network (RNN) demonstrated that the spatiotemporal information can be beneficial for individual identifications (Chen & Hu, 2018; L. Wang, Li, Chen, & Hu, 2019). The initial work applied a fully connected model to extract spatial features from ROI data and used RNN for temporal evolutions (Chen & Hu, 2018). In a follow-up study, spatial features among connectomic ROIs were extracted by convolutional layers (L. Wang et al., 2019). It showed that the identification accuracy increased with a larger number of input frames. The superior performance of the latter study suggests that proper convolution between ROIs for spatial features provides extra boosts for identification accuracy. However, it may be suboptimal to apply convolutions based on the predefined neighborhood that is defined by the functional atlas.

### Graph Convolutional Networks

Motivated by breakthroughs of deep learning on grid data, efforts have been made to extend CNN to graphs, a natural way to represent many forms of data including fMRI data. Two categories of graph convolutional networks (GCNs)—spectral GCNs and spatial GCNs—have been proposed. For spectral GCNs, graphs can be decomposed into spectral bases associated with graph-level information according to spectral graph theory (Bruna, Zaremba, Szlam, & LeCun, 2013). In contrast, spatial GCNs imitate the Euclidean convolution on grid data to aggregate spatial features between neighboring nodes. Although spectral GCNs have achieved great success on both structural and functional MRI applications (Gopinath, Desrosiers, & Lombaert, 2019; Hong et al., 2019; Ktena et al., 2018; Parisot et al., 2017), spatial models are preferred over the spectral ones because of their efficiency, generalization, and flexibility (Monti et al., 2017; Wu et al., 2019; Zhang, Cui, & Zhu, 2018), and they have gained increasing interest in the community (Azevedo, Passamonti, Liò, & Toschi, 2020; Gadgil et al., 2020).

In this paper, we present a connectivity-based graph convolutional network (cGCN),a spatial GCN architecture, for fMRI analysis. The graph representation was defined with the *k*-nearest neighbor (k-NN) graph based on FC matrix. Convolutions were performed on graphs rather than on an image/ROI grid, which allows us to efficiently extract connectomic features of the underlying brain activity. The present model (L. Wang, 2020; https://github.com/Lebo-Wang/cGCN_fMRI) has been applied in two scenarios, and the results indicate that cGCN is effective for fMRI analysis compared with traditional deep learning architectures.

## MATERIALS AND METHODS

### cGCN Overview

**Figure 1.**
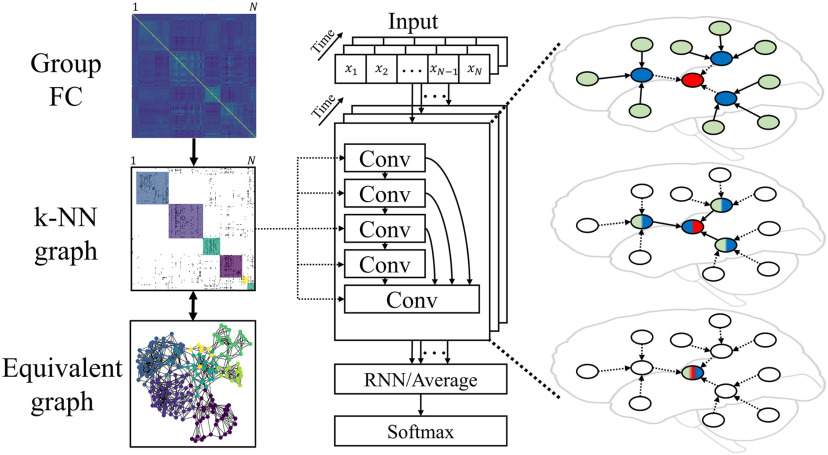
Overview of cGCN. On the left, the graph definition for cGCN was based on the group FC from all training data, which can be further simplified as a *k*-nearest neighbors (*k*-NN) graph with binarized edges. In the middle, the cGCN architecture consisted of 5 convolutional layers. The convolutional neighborhood was defined by the shared *k*-NN graph across convolutional layers, time frames, and subjects. The recurrent neural network (RNN) layer (or the temporal average pooling layer) obtained latent representations from all frames. The final classification was achieved by the Softmax layer. On the right, an intuitive illustration of the spatial graph convolution showed the information aggregation between neighboring nodes.

The overview of our cGCN architecture is shown in Figure 1. ROIs were considered to be graph nodes with the blood oxygen level–dependent (BOLD) signals of each frame as their attributes. Convolutions were performed within neighbors defined by the *k*-NN graph based on the groupwise FC matrix. Specifically, we first obtained the individual FC matrices on training data and averaged them for the group FC matrix. Based on that, a *k*-NN graph was generated by retaining only the top *k* edges in terms of their connectivity strength (i.e., average correlation coefficient) for each node. The FC-based *k*-NN graph was used to guide the convolutional operations with functional connectivity–based neighborhoods. For simplicity, the same graph was shared by all subjects and at all time frames. Although each node had a few neighbors, the convolution field of each layer was extended by stacking multiple convolutional layers in the architecture. Between convolutional layers, skip connections were added from the prior convolutional layers to the last one, providing multilevel feature fusion for classification and accelerating the model training by alleviating the vanishing gradient problem. Outputs from the convolutional layers were followed by an RNN (or a temporal average pooling) layer to generate temporal evolutions by combining spatial representations from all frames. A Softmax layer was used at the end for the final classification.

### Graph Construction

We represented each frame of fMRI data with a graph. A shared graph structure that reflects the intrinsic functional connectivity was derived from the group FC matrix (Petersen & Sporns, 2015), *based on training data only*. The ROIs were considered to be nodes of the graph, and the FC connections were considered edges of the graph. To reduce the total number of edges in the graph, a *k*-NN graph was obtained by keeping only the top *k* connectivity neighbors for each node in terms of the connectivity strength; *k* is the hyperparameter related to the topological structure of graphs, which controls the sparsity of the graph. To evaluate the effect of *k*, different values of *k* (3, 5, 10, and 20) were assessed in our experiments.

### The Edge Function of cGCN

The *k*-NN graph 𝒢 = (𝒱, 𝓔) comprises nodes 𝒱 = {1, …, *N*} and edges 𝓔 ⊆ 𝒱 × 𝒱. Edge (*i*, *j*) represents the directed edge from *ROI*_*i*_ to *ROI*_*j*_. To explicitly model the spatial pattern between ROIs, we chose EdgeConv, an asymmetric edge function (Y. Wang et al., 2018), as the convolutional operation for our cGCN convolutional layers:

$$x_{i}^{\prime }=\underset{j:(i,j)\in 𝓔}{max}h_{\Theta }(x_{i}∥x_{j}-x_{i}),$$

where *x*_*i*_ was the BOLD signal of the central node i and *x*_*j*_ was that of a connected neighbor node *j*. In addition to original features from the central node, the node value difference *x*_*j*_ − *x*_*i*_ was also appended as the complementary feature. *h*_Θ_ represents trainable weights Θ with a nonlinear activation function *h*, which was implemented by the multilayer perceptron (MLP) with the rectified linear unit (ReLU). The feature-wise convolution output, $x_{i}^{\prime }$, was the maximal activation from its top *k* neighbors. In this way, spatial features between connectivity-based neighbors were modeled, including the node activity and the coactivation pattern.

### Experiments and Settings

Two supervised classification experiments were carried out to evaluate the performance of our proposed architecture. In the first experiment, we used the “100 unrelated subjects” dataset (54 females, age: 22–36) released by the Human Connectome Project (HCP) and aimed to identify them based on their rs-fMRI data. Each subject had four rs-fMRI sessions (each with 1,200 volumes) scanned on two days (Van Essen et al., 2013). The fMRI data were preprocessed by the HCP minimal preprocessing pipeline (Glasser et al., 2013) and denoised by ICA-FIX (Salimi-Khorshidi et al., 2014) to remove spatial artifacts and motion-related fluctuations. Surface-based registration was performed with the MSM-ALL method (Robinson et al., 2014). We utilized 236 ROIs based on the Power atlas (Power et al., 2011). The two sessions from Day 1 were employed as the training dataset, and the two sessions from Day 2 were used as the validation dataset and test dataset, respectively. The best model was chosen according to the best validation accuracy and final identification accuracy was assessed on the test dataset. For the training and validation datasets, fMRI time courses were cut into 100-frame clips. The final identification performance was measured with different numbers of frames (from 1,200 frames to a single frame) on the test dataset. To evaluate the contribution of the connectivity-based neighborhood for convolutions, we also ascertained the performance of the same cGCN architecture with random graphs (random GCNs).

In the second experiment, we used cGCN to classify ASD patients from healthy controls on the ABIDE (Autism Brain Imaging Data Exchange) dataset (Di Martino et al., 2014). There are 1,057 subjects (525 ASD subjects and 532 neurotypical controls) from 17 imaging sites. All fMRI data were preprocessed by the Connectome Computation System pipeline (Zuo et al., 2013), with bandpass filtering (0.01–0.1 Hz) and without global signal regression. The Craddock 200 atlas (Craddock, James, Holtzheimer, Hu, & Mayberg, 2012) was utilized to extract ROI signals. We adopted both leave-one-site-out and tenfold cross-validations. For the leave-one-site-out cross-validation, data from each site were independently tested with the model trained on data from other sites, which evaluated the model on heterogeneous datasets considering the site-specific variation. The tenfold cross-validation mixed all data together and split them into different folds by keeping the proportions of sites and diagnostic groups across folds. The site-specific heterogeneity was overlooked by the random partition of subsamples.

We carried out the two experiments in Keras (Chollet, 2015) using TensorFlow as the back end (Abadi et al., 2016). Adam optimizer was applied to update model weights with adaptive learning rates (Kingma & Ba, 2014). Stepwise learning rate decay was also used if the validation accuracy stopped increasing, with the smallest learning rate of 1e-6. The model was evaluated on the validation dataset after each training epoch, and the model parameters were saved only if better validation accuracy was achieved. During training, the L2 regularization was used to avoid overfitting. The final performance was reported with the highest accuracy among different L2 values (0.1, 0.01, 0.001, and 0.0001).

### Visualization

The occlusion method (Zeiler & Fergus, 2014) was utilized to visualize the important ROIs for classification in both experiments. With trained model parameters, each ROI of input data was zeroed out one at a time, and the performance degradation under the same model configuration was considered as the contribution of the ROI to the classification task. The occlusion pattern was mapped onto the cortical surface for visualization. We projected ROI data to the cortical surface and obtained the surface renditions. For the individual identification task, the occlusion pattern was generated and averaged on the test dataset. For ASD classification, we utilized the pretrained models from the leave-one-site-out cross-validation and averaged the pattern of performance degradation on each leave-out dataset to get the visualization pattern.

## RESULTS

### Performance Related to the Number of Neighbors

**Figure 2.**
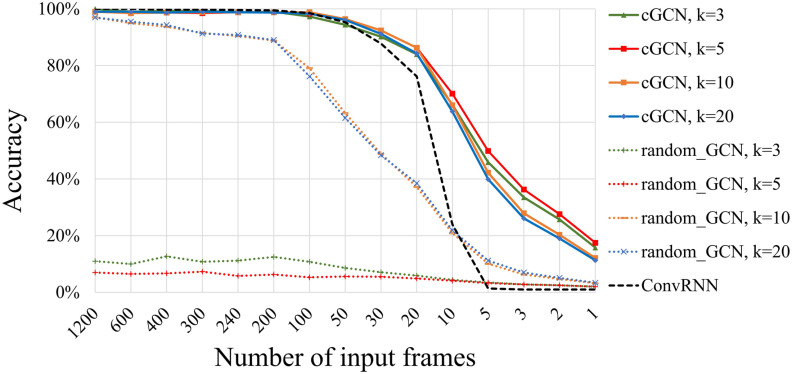
Performance on individual identification regarding the number of neighbors and the number of input frames. The highest mean classification accuracy was achieved with *k* = 5. With 20 frames or fewer as input data, cGCN achieved significantly higher classification accuracy compared with random GCNs and the convolutional RNN (ConvRNN) model in our prior study (L. Wang et al., 2019). Particularly with 5 frames, cGCNs obtained the identifying accuracy of over 49% with *k* = 5, which was much higher than random GCNs and ConvRNN. It demonstrated that spatiotemporal features from each frame of fMRI data was successfully extracted by cGCNs for the individual identification task.

The hyperparameter *k* determines the number of edges in graphs. The classification performance was evaluated for a range of *k* values (3, 5, 10, and 20). In Figure 2, we show the performance of individual identification. The cGCN with *k* of 5 achieved the highest identification accuracy on average. Increasing the *k* value beyond 5 (*k* = 10 or 20) led to diminished performance. The performance of random GCNs was significantly lower than cGCN models, especially for small *k* values (*k* = 3 or 5).

**Figure 3.**
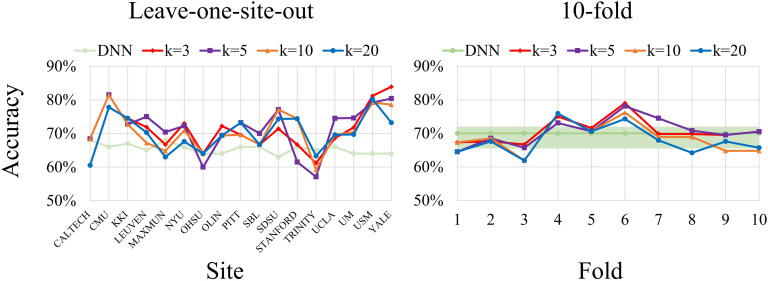
Performance on ABIDE datasets with the leave-one-site-out cross-validation and the tenfold cross-validation. The best average accuracy with the leave-one-site-out cross-validation on ABIDE dataset was 71.6% (min: 57.1%, max: 81.5%) when *k* = 5. As a comparison, the DNN model achieved mean accuracy of 65.4% (min: 63%, max: 68%; Heinsfeld et al., 2018). Except for some imaging sites (CALTECH, MAX_MUN, OHSU, and TRINITY), cGCNs obtained distinct performance improvement compared with the DNN model. The best average accuracy with the tenfold cross-validation was 70.7% (min: 66.7%, max: 79.0%) when *k* = 3, which outperformed the DNN model of 70% (min: 66%, max: 71%).

For ASD classification, the performance with leave-one-site-out and tenfold cross-validation are depicted in Figure 3. With the leave-one-site-out cross-validation, the highest mean accuracy was 71.6% when *k* = 5, and the lowest mean accuracy was 70.1% when *k* = 20. For the tenfold cross-validation, the highest mean accuracy was 70.7% when *k* = 3, and the lowest mean accuracy was 68.0% for cGCN with *k* = 20. On both cross-validations, cGCN with large *k* values (*k* = 10 or 20) had relatively lower classification accuracy.

### Performance Related to the Number of Input Frames

For the individual identification application, we evaluated cGCNs and random GCNs with different numbers of frames as inputs. With cGCN models, the improved performance of individual identification was shown with increasing numbers of fMRI frames. As shown in Figure 2, cGCN achieved better performance with a larger number of input frames, and the performance gradually saturated at approximately 100 frames. Random GCNs exhibited a similar pattern for large *k* values (*k* = 10 or 20), but with accuracy much lower than that of cGCNs. However, the performance of convolutional RNN (ConvRNN) dropped dramatically with reducing frames, close to the random guess (1%) with fewer than 5 frames. In contrast, cGCN still obtained an identifying accuracy of > 49% with 5 frames when *k* = 5.

**Figure 4.**
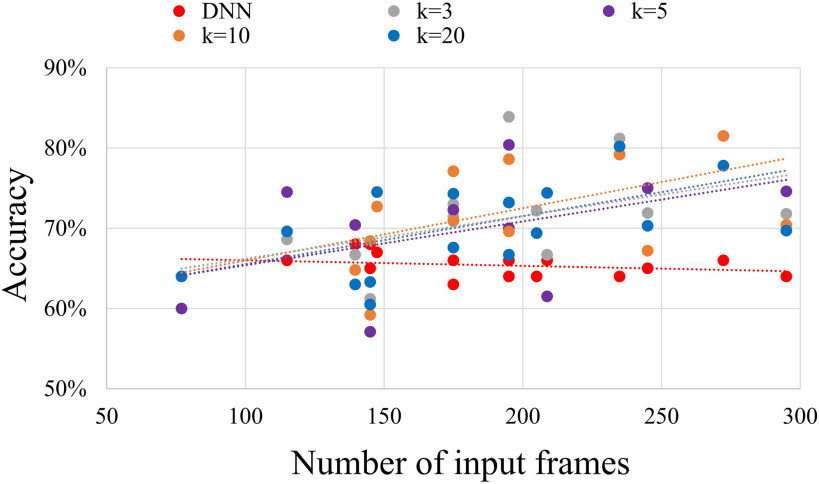
The relationship between the classification accuracy and the number of frames as inputs for the ASD classification task with the leave-one-site-out cross-validation. The average classification accuracy increased linearly with the number of input frames, while the number of input frames did not affect the classification accuracy for the deep neural network model with FC matrices as inputs (Heinsfeld et al., 2018).

Similarly, for the ASD classification task shown in Figure 4, better classification accuracy can be achieved with a larger number of input frames for different imaging sites in the leave-one-site-out cross-validation. As a contrast, the number of input frames did not significantly affect the classification accuracy of the deep neural network (DNN) model, in which FC matrices were used as inputs without considering the temporal dimension (Heinsfeld et al., 2018).

### Comparison

For the individual identification task, cGCN achieved an accuracy of 98.8% with *k* = 10 when the number of input frames was fixed to 100. It outperformed several prior architectures, including the RNN model of 94.4% (Chen & Hu, 2018), the ConvRNN model of 98.5% (L. Wang et al., 2019), and the traditional correlation method of around 70% (Finn et al., 2015). In particular, cGCNs showed overwhelming superiority over the traditional CNN model with fewer than 20 frames. As shown in Figure 2, the previous ConvRNN model with only 5 frames of input data led to an accuracy close to the random guess (1%).

For the ASD classification task with the leave-one-site-out cross-validation, the best mean classification accuracy was 71.6% (min: 57.1%, max: 81.5%) when *k* = 5 as shown in Figure 3. Except for some imaging sites (CALTECH, MAX_MUN, OHSU, and TRINITY), our cGCNs obtained significant performance improvement compared with the DNN model, whose mean accuracy was 65.4% (min: 63%, max: 68%; Heinsfeld et al., 2018). With the tenfold cross-validation, our best average performance was 70.7% (min: 66.7%, max: 79.0%) when *k* = 3, which is better than the RNN model of 68.5% (Dvornek, Ventola, Pelphrey, & Duncan, 2017) and the DNN model of 70% (min: 66%, max: 71%; Heinsfeld et al., 2018).

### Visualization

**Figure 5.**
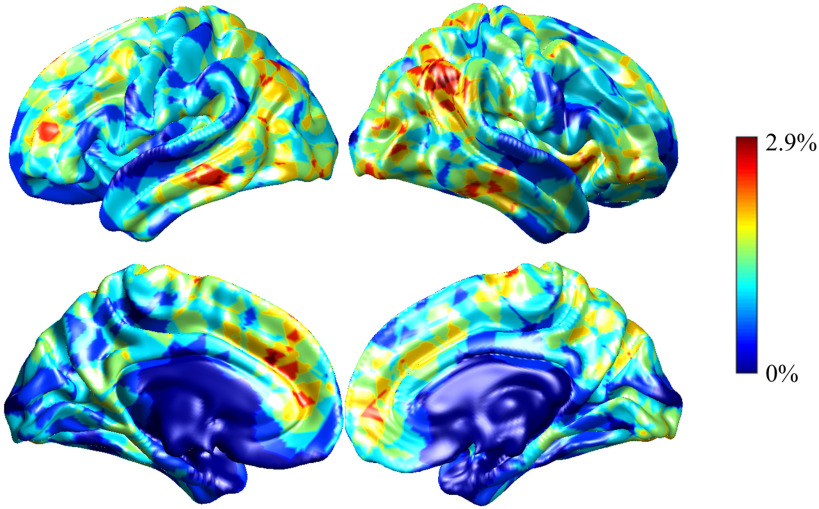
Visualization of the performance degradation through the single-ROI occlusion for the individual identification. The red region reflected large performance degradation if corresponding ROIs were occluded. The performance degradation reflects the relative contribution of each ROI. When *k* = 20, the smallest performance drop was only 0.6%, while the largest performance drop of 2.9% was obtained with *k* = 3. Default mode network, frontoparietal network, and visual network contributed more to the individual identification.

**Figure 6.**
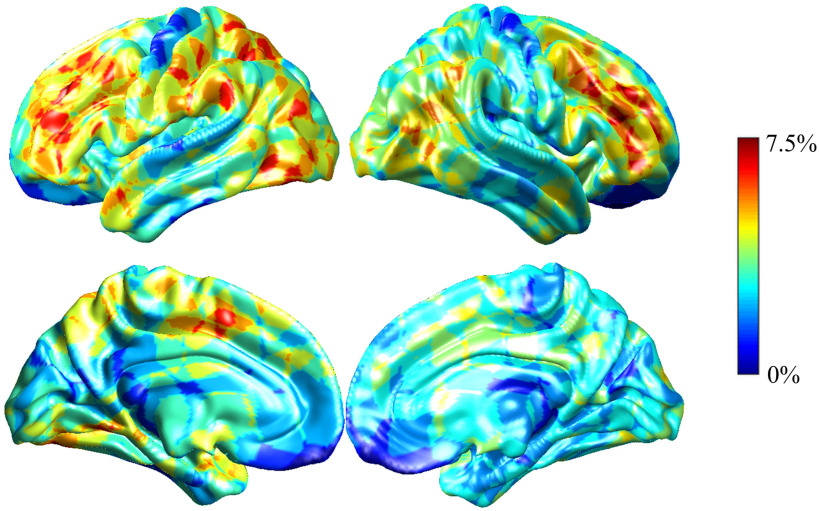
Visualization of the performance degradation through the single-ROI occlusion for the ASD classification. We averaged performance drop on all models used in the leave-one-site-out cross-validation. The maximum performance drop was 7.5% when *k* = 3, and the smallest performance drop was 6.0% when *k* = 20. The color of the region reflected the performance degradation if corresponding ROI was occluded. The salient regions related to the ASD classification included frontoparietal network, default mode network, and ventral attention network.

We applied the occlusion method to identify informative regions for two classification tasks and visualized them in Figures 5 and 6. For the individual identification task, the smallest accuracy degradation was 0.6% when *k* = 20 and the largest accuracy drop was 2.9% when *k* = 3. Significant performance degradation was observed when some ROIs were individually occluded, while some regions did not suffer from any performance degradation. The salient regions for cGCN models with different *k* values were similar. The significant resting-state networks were default mode network (DMN), frontoparietal network (FPN), as well as visual network (VN).

The visualization of cGCN for the ASD classification task is shown in Figure 6. The largest performance drop was 7.5% when *k* = 3, and the smallest performance drop was 6.0% when *k* = 20. The salient regions for cGCN models with different *k* values were similar. The salient networks identified by cGCN included FPN, DMN, and ventral attention network.

## DISCUSSION

We presented cGCN architecture for fMRI analysis and applied cGCN on two classification tasks with rs-fMRI data, that is, the individual identification and classification of ASD, respectively. The superior performance compared with prior studies demonstrated that cGCN can effectively capture spatial features of fMRI data within connectomic neighbors.

A major contribution of the present work is that rather than performing convolution on structured image grids, ROI-based fMRI data are considered graphs based on FC, and cGCN is carried out between connectomic neighbors on the graph. Previous studies utilized fully connected layers for the spatial feature extraction and ignored the brain’s functional organization. In addition, it is difficult to train fully connected models with good generalization because of overfitting. Our prior paper demonstrated that it was feasible to capture spatial patterns on a small batch of ROIs from the same FC network by convolutions (L. Wang et al., 2019), although ROIs were artificially arranged in a predefined order according to the atlas. In the present work, the convolutional neighborhood was defined by the group-level FC matrix. Each ROI was involved in the convolution with its top connectivity-based neighbors, and high-level features were extracted by stacking convolutional layers hierarchically. The superior performance of cGCN over random GCN and previously used methods demonstrated that connectivity-based neighborhood for convolutions was a significant advantage for the cGCN architecture.

We adopted the *k*-NN graph rather than hard thresholding FC to reduce the size of the neighborhood and effects of noise in connectivity (Liu, Nalci, & Falahpour, 2017; Murphy & Fox, 2017). The advantages of *k*-NN graphs lie in the following aspects. First, *k*-NN graphs naturally have good local homogeneity with the same number of edges originating from each node, appropriate for convolutions. Second, hierarchical feature extraction can be easily achieved with stacking layers under the guidance of the *k*-NN graph.

Like the convolutional kernel size in the traditional CNN models, *k* explicitly determines the convolutional region on graphs, as well as the computational complexity for the cGCN model. We tested the performance of our cGCN architecture with different *k* values. The best performance of the individual identification was achieved when *k* = 5, while the highest classification accuracy on ASD with two types of cross-validations was achieved when *k* = 3 or 5. For both tasks, increasing *k* did not significantly improve the classification accuracy. One possible reason is that convolution on too many neighbors might fail to generate local features with good generalization. The same situation also happened in the modeling of 3D objects (Y. Wang et al., 2018). Therefore, we suggest a *k* value of 5 or less for good performance, although the exact optimal value may be application dependent.

Unlike *k*, increasing the number of input frames significantly improved the performance for both tasks. This effect was especially obvious with a few input frames in the beginning and gradually leveled off with over 100 frames for individual identification. This result indicates that cGCN can efficiently extract spatial patterns between functionally “adjacent” nodes from each frame of fMRI data in spite of substantial intersubject and intersession variabilities that arise because of hardware variations, and physiological variations (McGonigle et al., 2000). Thus, it is beneficial to keep the temporal axis of the fMRI data and apply framewise feature extraction based on fMRI time course rather than the FC matrix.

There are some appealing properties by using the asymmetric edge function for convolutions according to Y. Wang et al. (2018). First, the spatial features of both global information *x*_*i*_ and local interactions *x*_*j*_ − *x*_*i*_ are captured by the asymmetric edge function. Second, the long-distance characterization of spatial features between multi-hop neighborhoods can be captured by the stacked convolutional structures, based on the topological structure of the graph.

Furthermore, the single-ROI occlusion experiments revealed the salient regions related to individual identification and classification of ASD tasks, which are in agreement with those seen in previous studies (Finn et al., 2015; Heinsfeld et al., 2018; Nielsen et al., 2013; L. Wang et al., 2019). For the individual identification task, the performance drop was much smaller than that of the ConvRNN model (L. Wang et al., 2019), suggesting that the graph-based representation of fMRI data builds robust relationships between ROIs, and missing values of any ROI might be compensated for by its functional neighbors based on the topological structure of graphs.

Some limitations should be noted for future work. First, the graph topology is invariant from layer to layer, frame to frame, and subject to subject. Considering the significant variability in FC, future work shall adopt dynamic update across convolutional layers, time frames, and subjects. Second, the graph in the present work reflects the brain’s organization only from the functional perspective. Other image modalities, such as diffusion MRI, could be utilized to build graphs that reflect the brain’s structural connectivity. Third, intuitive visualization of temporal features captured by cGCN is difficult. This warrants further investigation in future studies.

## CONCLUSION

In this paper, we describe a connectome-defined neighborhood for graph convolution to extract connectomic features from rs-fMRI data for classification. Our model allows for spatial feature extraction within connectomic neighborhoods rather than Euclidian ones. Significant improvement on individual identification and ASD classification tasks suggests that the cGCN model is effective in capturing connectomic features from fMRI data and is promising for fMRI analysis.

## AUTHOR CONTRIBUTIONS

Lebo Wang: Conceptualization; Methodology; Software; Validation; Visualization; Writing - Original Draft. Kaiming Li: Formal analysis; Methodology; Writing - Original Draft; Writing - Review & Editing. Xiaoping Hu: Conceptualization; Formal analysis; Methodology; Project administration; Resources; Writing - Original Draft; Writing - Review & Editing.

## TECHNICAL TERMS

rs-fMRI: Resting-state functional magnetic resonance imaging.
FC: Functional connectivity.
CNN: Convolutional neural network.
ROI: Region of interest.
ASD: Autism spectrum disorder.
RNN: Recurrent neural network.
GCN: Graph convolutional network.
cGCN: Connectivity-based graph convolutional network.
*k*-NN: k-nearest neighbors.
BOLD: Blood oxygen level–dependent.
